# PACCMIT/PACCMIT-CDS: identifying microRNA targets in 3′ UTRs and coding sequences

**DOI:** 10.1093/nar/gkv457

**Published:** 2015-05-06

**Authors:** Miroslav Šulc, Ray M. Marín, Harlan S. Robins, Jiří Vaníček

**Affiliations:** 1 Laboratory of Theoretical Physical Chemistry, Institut des Sciences et Ingénierie Chimiques, Ecole Polytechnique Fédérale de Lausanne (EPFL), CH-1015 Lausanne, Switzerland; 2 Fred Hutchinson Cancer Research Center, Seattle, WA 98109, USA

## Abstract

The purpose of the proposed web server, publicly available at http://paccmit.epfl.ch, is to provide a user-friendly interface to two algorithms for predicting messenger RNA (mRNA) molecules regulated by microRNAs: (i) PACCMIT (Prediction of ACcessible and/or Conserved MIcroRNA Targets), which identifies primarily mRNA transcripts targeted in their 3′ untranslated regions (3′ UTRs), and (ii) PACCMIT-CDS, designed to find mRNAs targeted within their coding sequences (CDSs). While PACCMIT belongs among the accurate algorithms for predicting conserved microRNA targets in the 3′ UTRs, the main contribution of the web server is 2-fold: PACCMIT provides an accurate tool for predicting targets also of weakly conserved or non-conserved microRNAs, whereas PACCMIT-CDS addresses the lack of similar portals adapted specifically for targets in CDS. The web server asks the user for microRNAs and mRNAs to be analyzed, accesses the precomputed *P*-values for all microRNA–mRNA pairs from a database for all mRNAs and microRNAs in a given species, ranks the predicted microRNA–mRNA pairs, evaluates their significance according to the false discovery rate and finally displays the predictions in a tabular form. The results are also available for download in several standard formats.

## INTRODUCTION

MicroRNAs (miRNAs) form one of the classes of small non-coding RNA molecules that have completely transformed our understanding of gene regulatory networks. It is now well established that these short (∼20–23 nt long) endogenous non-coding RNA molecules play an essential role in development, cancer, viral infection and many other important biological processes (1). The miRNAs act via binding to the target messenger RNA (mRNA) (2), which can either lead to the repression of the translation of an mRNA into a protein or even trigger the degradation of this mRNA (3–5). Since a large fraction of mammalian mRNAs appear to be targeted by miRNAs (6), identification of functional miRNA–mRNA pairs is of paramount importance and provides a challenge to both experiment and theory. Although a full and direct experimental validation of many candidate miRNA-target interactions remains tedious, there exist several indirect methods such as luciferase-reporter, mRNA expression (7,8), protein expression (7,8) or cross-linking immunoprecipitation (CLIP) assays (9,10), the last three of which permit a high-throughput implementation. The high cost in time, personnel and material of these experimental techniques, however, advocates the development of computational algorithms tailored for identification of miRNA targets in order to at least partially narrow down the experimental search. The strategy is quite straightforward in plants or in case of another class of small RNA molecules—the small interfering RNAs (siRNAs): there, a near-perfect complementarity is required with the targeted mRNA. Since the length of a typical miRNA/siRNA is at least 20 nt, it is rather unlikely that the complementary sequence would appear anywhere in the genome by chance, leading to simple target prediction algorithms based only on sequence complementarity. However, the mammalian miRNAs bind to target mRNAs only partially, typically via a ‘seed region’ of six to eight consecutive nucleotides located at the 5′ end of the miRNA. Although the seed is the most important determinant of a functional miRNA–mRNA interaction, a simple back-of-the-envelope calculation shows that scanning the transcripts for regions complementary to a 6-mer seed would predict that each miRNA regulates the 3′ UTR of every fifth transcript (assuming a typical 3′ UTR length of about 1000 nt and equal probability of the 4 nt) and the coding sequence of even more transcripts. To increase the precision of their predictions, most algorithms have incorporated additional requirements beside the complementarity to the seed.

While some algorithms consider the miRNA sequence surrounding the seed and add empirical rules based on known functional miRNA-target pairs, we developed two algorithms that still only consider the seed sequence of the miRNA, but evaluate the significance of the appearance of a seed match in the target sequence: PACCMIT (11–14) is designed for identifying miRNA targets primarily in 3′ UTRs, while PACCMIT-CDS (15) is adapted specifically to coding sequences. These algorithms, the details of which are given below, were successfully used, e.g. to predict miRNA roles in the latency and reactivation of herpesviruses (16,17) and the discovery of polymorphic miRNA target sites within the swine leukocyte antigen complex (18). Although there exist several web sites providing access to algorithms predicting conserved miRNA targets in the 3′ UTR, our web server for PACCMIT will fill a need for an accurate tool for predicting targets of weakly conserved miRNAs (12); likewise, a web server for PACCMIT-CDS will find its place among accurate tools designed specifically for predicting miRNA targets in the coding region.

### PACCMIT algorithm

The original algorithm (11,16) ranks predictions according to the over-representation of sites complementary to the miRNA seed (the so-called ‘seed matches’) with respect to a random background based on a Markov model. The precision is further increased by considering only conserved (11,16), partially accessible (12) (PACMIT, Prediction of ACcessible MIcroRNA Targets), or conserved and partially accessible (13,14) (PACCMIT) sites. Using partially accessible sites, i.e. sites containing an accessible 4-mer (19) in at least 20% of mRNA secondary structures improves the precision of predicted targets of weakly conserved miRNAs, while using conserved seed matches improves the predictions of targets of highly conserved miRNAs. In both cases, ranking by over-representation yields a significantly higher precision than the precision obtained by standard free-energy based methods (13). We have shown that the precision of PACCMIT can be further increased by requiring the partially accessible 4-mer to be located at the 3′ end of the seed match (14).

### PACCMIT-CDS algorithm

This algorithm (15) finds potential miRNA targets within CDS by searching for conserved motifs complementary to the miRNA seed region and ranking them according to over-representation with respect to a random background preserving both codon usage and amino acid sequence (20,21).

### Validation of the two algorithms

Precision and sensitivity of the two algorithms were evaluated by constructing validation data sets based on the binding sites reported in the PAR-CLIP (Photoactivatable-Ribonucleoside-Enhanced CLIP) experiments (9) and on the changes in the protein expression from proteomics experiments (7,8). These two validation approaches are complementary since they test very different aspects of miRNA-transcript targeting: while the PAR-CLIP data set provides direct information about physical binding between miRNA and mRNA, but does not say much about its biological functionality, the proteomics data sets reflect the effect of miRNAs on protein expression (i.e. function) but cannot distinguish direct from indirect effects. In both validation approaches, PACCMIT and PACCMIT-CDS performed extremely well, particularly as far as the precision of the top predictions is concerned (12,13,15).

The first validation approach employed data sets compiled from positive and negative interactions obtained in PAR-CLIP experiments (9). For the validation of PACCMIT, protein coding genes were considered to be true targets if their 3′ UTR was Argonaute (AGO)-bound and contained at least one seed match for any of the 100 most abundant miRNAs; altogether, 3698 such positive interactions were found. Negative interactions were defined as genes with no evidence of AGO binding in the entire transcript but harboring seed matches in their 3′ UTR. Given that negative interactions were much more abundant, only 3698 randomly chosen negative interactions were retained for the analysis in order to achieve a balance between positives and negatives. The validation data set for PACCMIT-CDS was built using similar principles; true targets were defined as genes whose coding region contained at least one seed match overlapping with an AGO-bound region. However, this time we considered only the 74 evolutionarily conserved miRNAs within the set of the 100 most abundant miRNAs. Thus, the consolidated data set for PACCMIT-CDS contained a total of 4376 interactions of which 2188 were positive and 2188 negative.

The second approach of validating PACCMIT and PACCMIT-CDS relied on the proteomics data of Baek et al. (7) and Selbach et al. (8), providing the protein fold changes (FCs) measured after overexpression of three and five conserved miRNAs respectively. In this case, miRNA-gene pairs with log_2_FC ≤ −0.2 were labeled as positive interactions (i.e. true targets) while the remaining pairs were labeled as negative ones (false targets). Further details about preparation of validation data sets can be found in Refs. (13–15).

## Web SERVER

The web server is available for public use at http://paccmit.epfl.ch. In this section we explain the computational workflow of the web server, introduce its user interface and finally discuss the most important features of the current implementation.

The workflow can be summarized as follows: the user is first requested to select one of the two algorithms available, either PACCMIT (13) for transcripts targeted in the 3′ UTRs or PACCMIT-CDS (15) for transcripts targeted in the CDSs. Apart from this, it is possible to increase the precision of the selected algorithm by imposing conservation (available for both PACCMIT and PACCMIT-CDS) and/or accessibility (available for PACCMIT only) filters. In the next step, the user specifies the miRNAs and mRNAs to be analyzed. From this input, the web server identifies candidate miRNA-target pairs, ranks them according to a *P*-value, evaluates the statistical significance according to the false discovery rate based on the Benjamini–Hochberg procedure (22) and interactively returns a list of miRNA/mRNA pairs in a specified format discussed in more detail below.

Main references to the relevant literature as well as a brief explanation of the key concepts used in PACCMIT and PACCMIT-CDS can be found in the ‘Help’ section of the web server. Finally, the ‘Tutorial’ section provides a minimalistic example demonstrating a typical use of the web server. These sections are directly accessible from the horizontal menu located in the header of the front page.

### Web-server interface

The main user interface of the web server can be reached via the link ‘Predictions’ from the horizontal menu. This link opens a simple four-step wizard which asks the user to provide the following information:

1. Algorithm & database
  1. Selection of the algorithm: either PACCMIT for targets in 3′ UTRs or PACCMIT-CDS for targets in CDSs.
  2. Possibility to invoke accessibility (PACCMIT only) and/or conservation (PACCMIT and PACCMIT-CDS) filters in order to increase precision (and hence, usually, to decrease sensitivity).
    As for accessibility, one can choose one of three options: (1) No accessibility filter at all—this option yields the highest sensitivity; (2) a ‘loose’ version, which allows the accessible 4-mer to lie anywhere within the seed match; or (3) a ‘strict’ version, which yields the highest precision by requiring the partially accessible 4-mer to lie at the 3′ end of the seed match.
  3. Selection of the genome, assembly, and track of interest.
2. miRNAs—miRNA accession numbers can be pasted directly into the web site or uploaded as a text file. Both input sources are merged and any duplicities are automatically removed.
3. mRNAs—transcript IDs are provided in the same fashion as the accession numbers of miRNAs in the previous step. Note that the particular format of these IDs depends on step 1.c.
4. Output
  1. Specification of the maximum number of returned miRNA/mRNA pairs. If the number of pairs happens to be greater than 10 000, the output will be automatically written to a CSV file, overriding the choice in step 4.c.
  2. Imposition of the false discovery rate (FDR) level $0 \le \alpha \le 1$ in the spirit of the Benjamini–Hochberg procedure (22); only predictions (i.e. miRNA/mRNA pairs) significant at that particular level are returned.
  3. Selection of the output format. Returned predictions can be either displayed in a table form within the browser or exported to a Microsoft Excel spreadsheet or standard CSV file.

In the output, the predictions are ranked according to the *P*-value that the observed number of conserved and/or accessible seed matches would appear in the target sequence by chance. In order to evaluate statistical significance, the ‘adjusted’ *P*-values are also shown. These adjusted *P*-values are calculated with an analog of the R function call p.adjust(x, method = ‘BH’) (23) based on the Benjamini-Hochberg procedure (22). Finally, for every predicted miRNA/mRNA pair, we also include a comma-delimited list of the corresponding seed match positions (1-based). Note that if there are multiple such positions for a given miRNA/mRNA pair, by design our algorithm is not able to determine which of them is more likely to be functional or whether more of them are likely to be functional. Our algorithm only predicts if the given mRNA as a whole is likely to be targeted by the given miRNA.

### Input data

The web server employs mRNA sequences as provided by the UCSC Table browser [http://genome.ucsc.edu/cgi-bin/hgTables (24)] and miRNA sequences obtained from miRBase database [http://miRbase.org (25)].

### Practical notes

#### Algorithm & database

The current version of the web server supports the following algorithm and database combinations:

- PACCMIT:
  - NCBI36/hg18, Ensembl genes
  - GRCh38/hg38, Ensembl genes
  - GRCh38/hg38, RefSeq genes
- PACCMIT-CDS:
  - NCBI36/hg18, Ensembl genes

#### miRNAs

**Figure 1.**
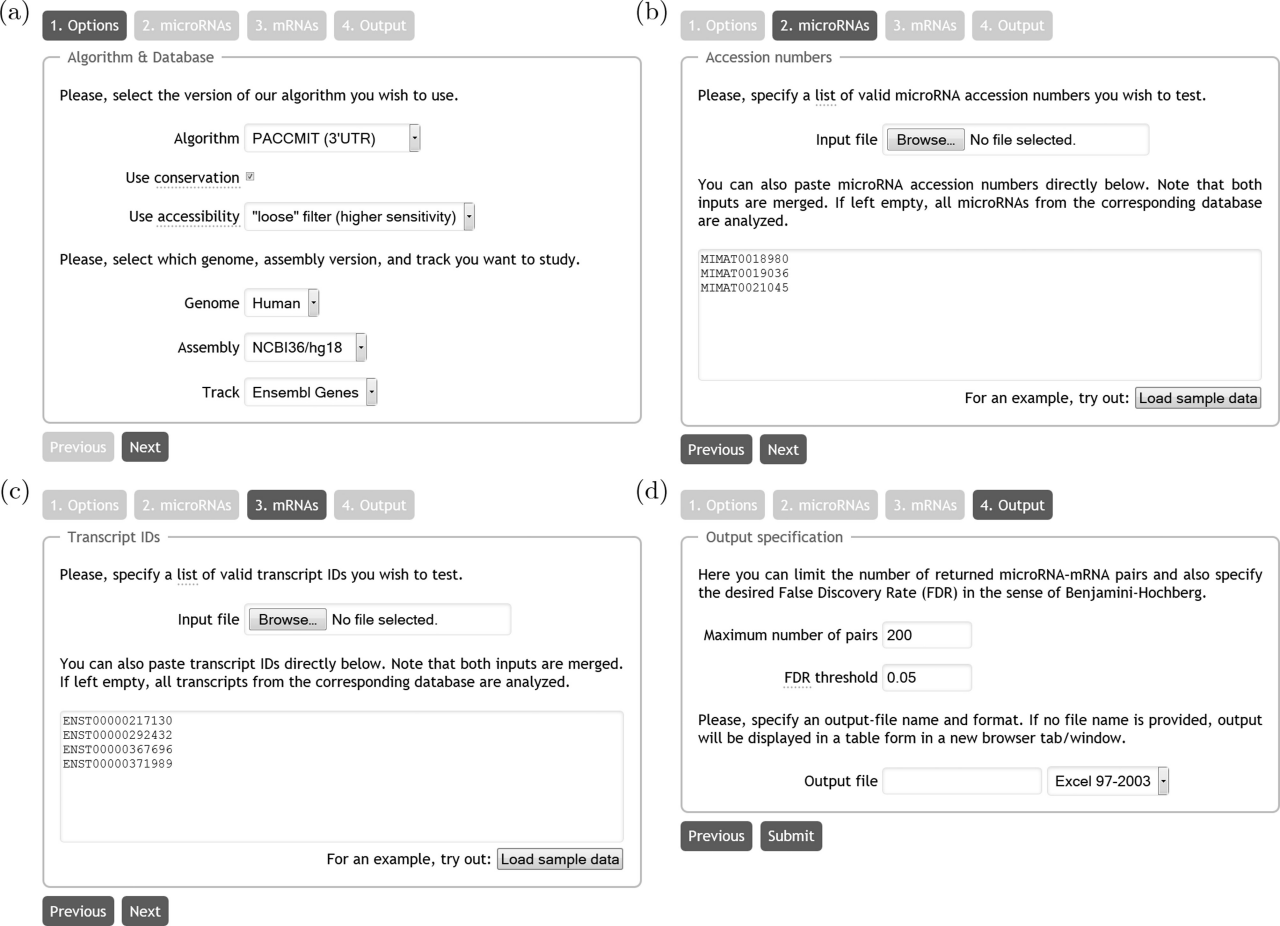
Overview of the main user interface of the PACCMIT/PACCMIT-CDS web server. (a) Selection of the algorithm and database. (b) Input of miRNAs of interest. (c) Input of mRNAs of interest. (d) Specification of the output parameters. For details see the main text.

Since the miRNA names are occasionally updated due to consolidation of the miRNA databases, to avoid problems, the web server identifies individual miRNAs by accession numbers, e.g. MIMAT0000076 instead of hsa-miR-21-5p. If the user does not provide any accession numbers, the web server will analyze all miRNAs available in the database. A short list of ‘example’ miRNAs can be loaded by clicking the ‘Load sample data’ button (see Figure 1b).

#### mRNAs

A particular choice of the track (Ensembl/RefSeq) determines the format of the transcript (mRNA) IDs, e.g. ENST00000371026 (Ensembl) or NM_024763 (RefSeq). Note that supplied Ensembl IDs should not contain a suffix denoting particular ‘version’; e.g. ENST00000371007.4 would not be recognized as a valid ID. As in the previous step, if no IDs are supplied, all available transcript IDs are processed. As for the miRNAs, the ‘Load sample data’ button loads a short list of ‘example’ mRNAs (see Figure 1c).

#### Output

The user can specify the name and format of the output file into which the miRNA target predictions (miRNA/mRNA pairs) will be saved. If no output file name is specified, these predictions are displayed within the browser. Current version of the web server supports the export to Microsoft Excel (version 97 and newer) and CSV files. While the exported CSV file only contains the predictions, both the HTML output (within the browser) and Excel spreadsheet contain additional information (see the following subsection for an example). In particular, the miRNA accession numbers and the mRNA transcript IDs from the input are divided into two distinct groups: The first group, called ‘Discarded’, contains items which were not found in the database, while the second group, called ‘Used’, contains items (miRNA accession numbers or mRNA transcript IDs) present in the database, and therefore considered in the search. Finally, the unique miRNAs and mRNAs (if any) appearing in the predictions are listed in tabular form, together with the miRNA sequence, gene name associated with the mRNA and some other information.

### Minimalistic example

In order to demonstrate a typical use of the PACCMIT/PACCMIT-CDS web server, we provide a minimalistic example in which potential miRNA targets in the 3′ UTRs are identified for a small set of miRNAs and mRNAs.

To this end, we employ the PACCMIT algorithm taking into account both the conservation and accessibility filters [see (13)]. As for the database, we consider the NCBI36/hg18 assembly and Ensembl genes. Moreover, we test only possible interactions between three miRNAs and four mRNAs.

Screenshots of the four steps of the wizard introduced above are shown in the four panels of Figure 1. The choice of the algorithm, additional filters and database (Step 1) is reflected in Figure 1a. Figure 1b then shows how to enter the desired miRNA accession numbers (MIMAT0018980, MIMAT0019036 and MIMAT0021045) while Figure 1c contains the pasted mRNA transcript IDs (ENST00000217130, ENST00000292432, ENST00000367696 and ENST00000371989). The format of the miRNA accession numbers (Figure 1b) is independent of the setup in Figure 1a. However, a particular choice of the ‘Track’ in Figure 1a directly determines the allowed format of the input (i.e. mRNA IDs) in Figure 1c. Finally, Figure 1d shows how to limit the maximum number of returned pairs to 200 (not relevant here since the theoretical maximum of returned miRNA/mRNA pairs is $3 \times 4 = 12$) and Benjamini–Hochberg FDR threshold to 5%.

**Figure 2.**
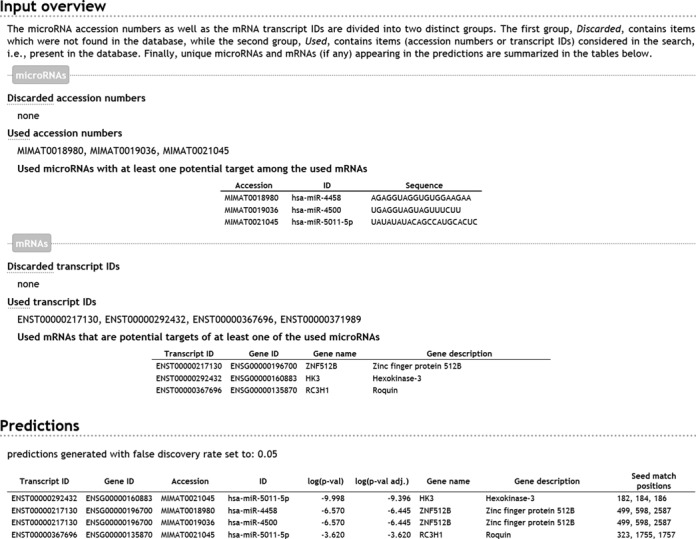
Example of the output generated by the PACCMIT/PACCMIT-CDS web server. This output corresponds to the input parameters from Figure 1 and discussed in the main text.

After the user clicks the ‘Submit’ button (Figure 1d) the web server processes the input and displays the output directly within the browser since the input field ‘Output file’ in Figure 1d is left empty. The structure of the output should, in the majority of modern browsers, closely resemble Figure 2. The top part of the output, labeled as ‘Input overview,’ shows which user-supplied miRNA accession numbers and mRNA IDs were discarded and which were used in the search (see subsection Practical notes). The top section also explicitly lists those used miRNAs from the input that have at least one potential target among the used mRNAs and those used mRNAs from the input that are potential targets of at least one used miRNA. By a potential target is meant an mRNA that contains a seed match (i.e. a 7-mer complementary to the seed of a given miRNA) satisfying the required accessibility and/or conservation filters. The web server automatically provides IDs and nucleotide sequences for the miRNAs as well as gene names/descriptions for mRNAs. In this particular example, we see that the transcript ENST00000371989 does not appear among ‘Used mRNAs that are potential targets of at least one of the used miRNAs’. In general, there can be two reasons for this: either (i) there is no (conserved and accessible) seed match for any of the miRNAs specified in the input—this is what happens in our example or (ii) the output restriction in Figure 1d is too stringent (i.e. ‘maximum number of pairs’ and/or ‘FDR threshold’ values are too low). Finally, the second, bottom part of the output, labeled ‘Predictions’, contains the identified miRNA/mRNA pairs ranked according to a statistical *P*-value, the most likely miRNA-target interactions having the smallest *P*-value. For each predicted pair, the web server also calculates the ‘adjusted *P*-value’ in the sense of Benjamini and Hochberg (22). This value is provided in order to address the problem of multiple hypotheses.

If the output is exported into Microsoft Excel, the generated spreadsheet contains seven sheets: three for an overview of the miRNAs (discarded accession numbers, used accession numbers and miRNAs with potential targets), three for an overview of the mRNA transcripts (discarded mRNA IDs, used mRNA IDs and potentially targeted mRNAs) and finally, the most important sheet containing the section ‘Predictions’ from Figure 2. In contrast, exported CSV files contain only the section ‘Predictions’.

### Citing the PACCMIT/PACCMIT-CDS web server

Should you wish to employ the proposed web server in your research, we would like to kindly ask you to cite this manuscript as well as one of the original publications (depending on the algorithm of interest), i.e. for targets in the 3′ UTR (PACCMIT algorithm):

1. (11) for PACCMIT with no filter or conservation filter alone,
2. (12) for PACCMIT with the ‘loose’ accessibility filter alone,
3. (13) for PACCMIT with both conservation and ‘loose’ accessibility filters,
4. (14) for PACCMIT with the ‘strict’ accessibility filter (alone or with conservation filter);

for targets in the CDS (PACCMIT-CDS algorithm): (15).

## CONCLUSIONS

In conclusion, we have presented a simple-to-use web server for accurate prediction of targets of both conserved and non-conserved miRNAs both in the 3′ UTR (PACCMIT) and in the coding sequences (PACCMIT-CDS). In the future, we plan to expand the functionality of the web server by incorporating more species as well as to keep the web server up-to-date by employing the current assemblies of the genomes already included. Finally, due to the flexibility of the two algorithms, in which ranking is independent of conservation and accessibility filters, it will be easy to improve the algorithms (and hence the web server) by incorporating new features based on experimental evidence that may become available over time.
